# Improving Access to Self-Expanding Metal Stents for Patients With Esophageal Cancer in Eastern Africa: A Stepwise Implementation Strategy

**DOI:** 10.1200/GO.20.00318

**Published:** 2021-01-15

**Authors:** Beatrice P. Mushi, Michael M. Mwachiro, Geoffrey Buckle, Bongani N. Kaimila, Gift Mulima, Violet Kayamba, Paul Kelly, Larry Akoko, Elia J. Mmbaga, Msiba Selekwa, Yona Ringo, Natalie Pritchett, Russell E. White, Mark D. Topazian, David E. Fleischer, Sanford M. Dawsey, Katherine Van Loon

**Affiliations:** ^1^ Muhimbili University of Health and Allied Sciences, Dar es Salaam, Tanzania; ^2^ Tenwek Hospital, Bomet, Kenya; ^3^ Global Cancer Program, Helen Diller Family Comprehensive Cancer Center, University of California, San Francisco (UCSF), San Francisco, CA; ^4^ University of Malawi College of Medicine, Blantyre, Malawi; ^5^ Kamuzu Central Hospital, Lilongwe Malawi; ^6^ University of Zambia School of Medicine, Lusaka, Zambia; ^7^ Muhimbili National Hospital, Dar es Salaam, Tanzania; ^8^ Division of Cancer Epidemiology and Genetics, National Cancer Institute, National Institutes of Health, Bethesda, MD; ^9^ Mayo Clinic, Rochester, MN; ^10^ Mayo Clinic, Phoenix, AZ

## Abstract

**PURPOSE:**

The eastern corridor of Africa is affected by a high burden of esophageal cancer (EC), with > 90% of patients presenting with advanced disease. Self-expanding metal stents (SEMS) have been previously reported as safe and effective for palliation of malignant dysphagia in resource-limited settings; however, access is limited throughout Eastern Africa.

**METHODS:**

In response to demand for palliative interventions for patients with dysphagia because of EC, the African Esophageal Cancer Consortium (AfrECC) partnered with the Clinton Health Access Initiative to improve access to SEMS in Eastern Africa. We undertook a stepwise implementation approach to (1) identify barriers to SEMS access, (2) conduct a market analysis, (3) select an industry partner, (4) establish regulatory and procurement processes, (5) develop endoscopic training resources, (6) create a medical device registry, and (7) establish principles of accountability.

**RESULTS:**

Following an evaluation of market demand and potential SEMS manufacturers, Boston Scientific Corporation announced its commitment to launch an access program to provide esophageal SEMS to patients in Tanzania, Kenya, Malawi, and Zambia at a subsidized price. Parallel regulatory and procurement processes were established in each participating country. Endoscopy training courses were designed and conducted, using the Training-of-Trainers model. A device registry was created to centralize data for quality control and to monitor channels of SEMS distribution. Principles of accountability were developed to guide the sustainability of this endeavor.

**CONCLUSION:**

The AfrECC Stent Access Initiative is an example of a multisector partnership formed to provide an innovative solution to align regional needs with a supply chain for a high-priority medical device.

## BACKGROUND

**FIG 1:**
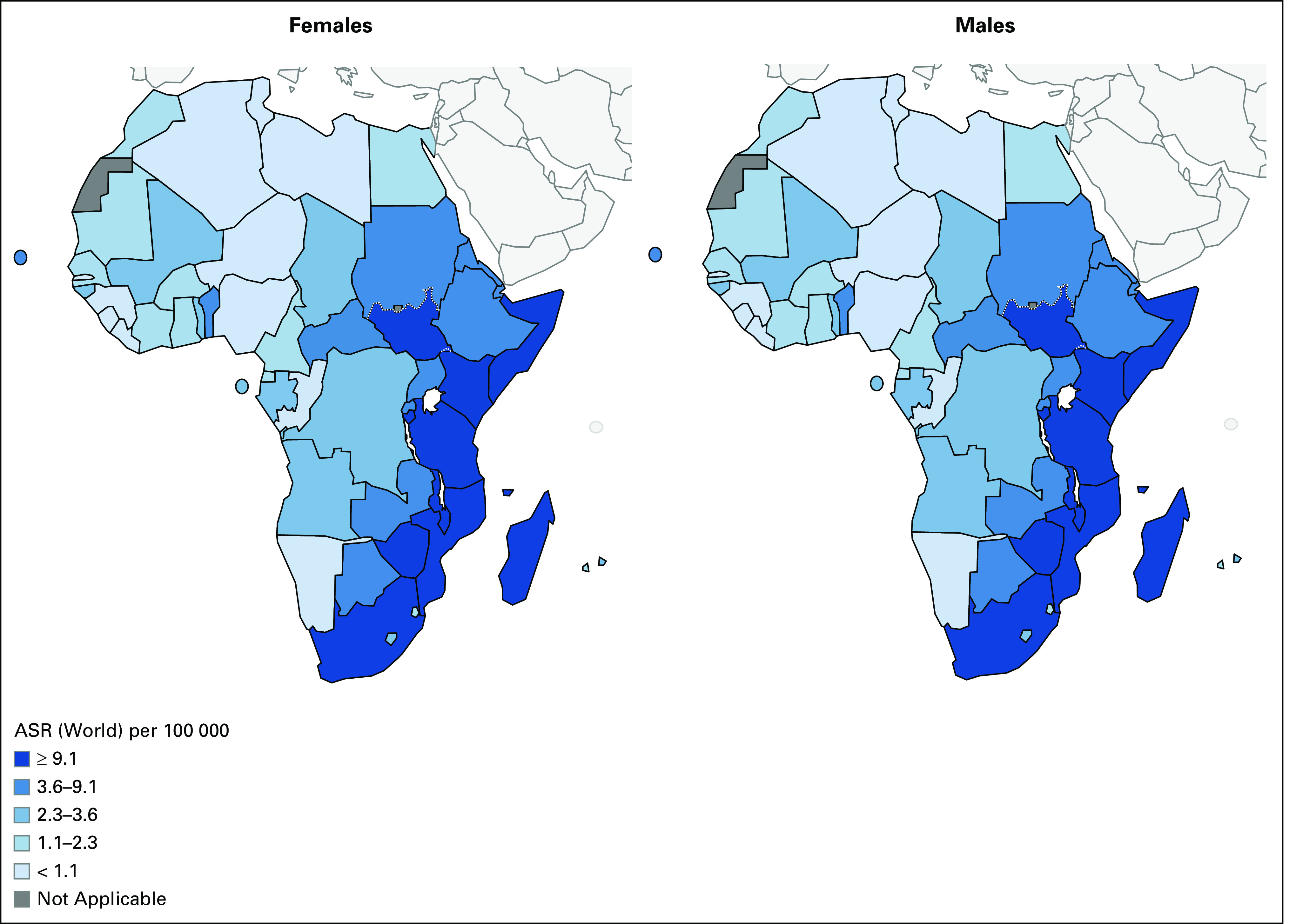
Map of estimated ASRs of esophageal cancer for males and females in Africa. ASR, age-standardized incidence rate. Reprinted with permission from Globocan (2018).^27^

Esophageal cancer (EC) is the seventh most common cancer and the sixth most common cause of cancer-related mortality worldwide.^1^ EC is characterized by wide geographic variations in incidence, and more than 80% of cases and deaths occur in developing countries.^1^ The eastern corridor of Africa, spanning from Ethiopia to South Africa, has been increasingly recognized for its disproportionately high burden of EC (Fig 1). Although the dominant subtype of EC in developed countries is adenocarcinoma, squamous cell carcinoma is the dominant subtype in high-incidence areas in developing countries, including countries throughout Eastern Africa.^2^

**CONTEXT**

**Key Objective**
The African Esophageal Cancer Consortium and the Clinton Health Access Initiative partnered to strategically address the need for access to self-expanding metal stents (SEMS) for palliation of malignant obstruction in patients with esophageal cancer in Eastern Africa.
**Knowledge Generated**
Through a stepwise implementation approach, we identified barriers to SEMS access, conducted a market analysis, selected an industry partner, established regulatory and procurement processes within each country, developed endoscopic training resources, created a medical device registry, and established principles of accountability.
**Relevance**
This is an example of a multisector partnership formed to provide an innovative solution to align regional needs with a supply chain for a high-priority medical device. Progress in closing the divide of availability of essential medical devices for patients with cancer in low-resource settings is needed. Implementation strategies require thoughtful coordination and formation of partnerships between industry, academia, health-care systems, and policy makers.

More than 90% of patients diagnosed with EC in Eastern Africa present with advanced disease, resulting in poor outcomes.^3,4^ Presentations with obstructive dysphagia are ubiquitous, and malnutrition is a major cause of morbidity and mortality. In a study conducted in Tanzania, of 430 patients who were treated with radiation for palliation of EC, 38% of patients died during or immediately following treatment.^3^ Health-care insurance in Eastern Africa is not widely subscribed, and cancer services are typically funded by a combination of government subsidy and out-of-pocket costs absorbed by patients. Although access to chemotherapy and radiation therapy is improving as a result of efforts to build oncology capacity, options for palliative and therapeutic management of advanced EC remain limited. Despite the high burden of EC in Eastern Africa and the need for a low-cost palliative interventions, self-expanding metal stents (SEMS) are not routinely available throughout the region, even at National Referral Centers.

Deployment of SEMS offers a nonsurgical approach to palliate severe dysphagia and improves nutritional support for patients who present with malignant obstruction.^5-9^ SEMS can be used as sole therapy or in combination with chemotherapy and/or radiation, with low rates of procedure-related and long-term complications.^10,11^ Tenwek Hospital, a faith-based community hospital in Kenya, became a high-volume referral center for patients with EC due to its ability to source SEMS at low cost and perform flexible GI endoscopies with SEMS placement, providing a model of care for patients with EC with dysphagia in sub-Saharan Africa.^12^ Between 1999 and 2008, 951 patients with inoperable EC underwent SEMS placement at Tenwek Hospital.^13^ A prospective case series from Tenwek reported that patients experienced immediate improvement in dysphagia symptoms, faced comparatively less financial burden, and were managed on an outpatient basis.

The African Esophageal Cancer Consortium (AfrECC) was established to address the burden of EC affecting Eastern Africa.^14^ As one of its goals, AfrECC aims to build capacity for and improve access to treatment and palliation. In 2016, AfrECC and the Clinton Health Access Initiative (CHAI) partnered to strategically address the need for access to SEMS in Eastern Africa. Herein, we aim to share the approach undertaken to introduce a new medical device in this region.

## METHODS

**FIG 2:**
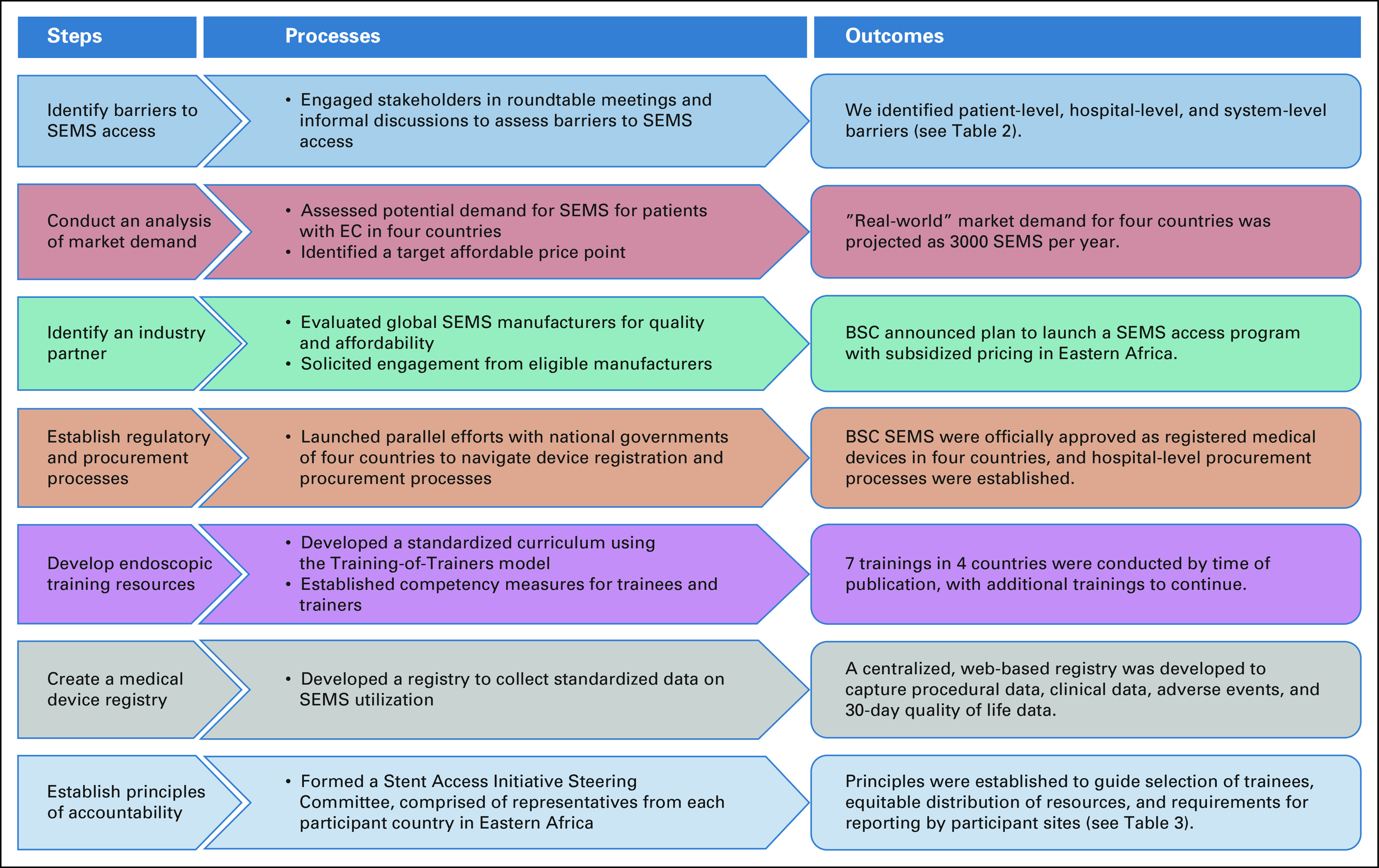
A framework for the development of the African Esophageal Cancer Consortium stent access initiative. BSC, Boston Scientific Corporation; EC, esophageal cancer; SEMS, self-expanding metal stents.

High-volume referral centers in Kenya, Tanzania, Malawi, and Zambia with preexisting endoscopic expertise and equipment were selected as sites for the initial phase of the intervention (Table 1). We undertook a stepwise implementation approach to (1) identify barriers to SEMS access, (2) conduct a market analysis, (3) select an industry partner, (4) establish regulatory and procurement processes, (5) develop endoscopic training resources, (6) create a medical device registry, and (7) establish principles of accountability (Fig 2).

**TABLE 1:**
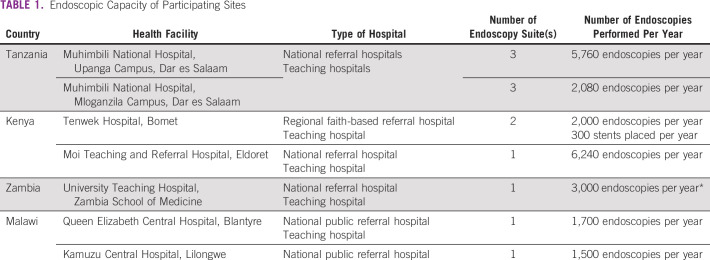
Endoscopic Capacity of Participating Sites

### Step 1: Identification of Barriers to Access

Barriers were defined as those factors obstructing timely access to health services necessary to achieve the best outcome.^15^ To identify barriers, an in-person needs assessment was conducted at each participating site. Information was gathered during discussions with hospital administrators, endoscopy unit administrators, endoscopists, and nurses.

We applied a multilevel framework to evaluate the interplay between individuals, hospitals, and health-care systems as determinants of barriers to SEMS access (Table 2). Patient-level barriers included challenges in accessing health-care facilities, low levels of health literacy, and prevailing stigmas around a cancer diagnosis. Hospital-level barriers pertained to the needs for adequate endoscopic capacity and training necessary to support SEMS placement. System-level barriers included prohibitive device costs and importation taxes, bureaucracy in regulatory and procurement processes, lack of regulatory enforcement to prevent illegal device distribution, and inadequate coverage of cancer services.

**TABLE 2:**
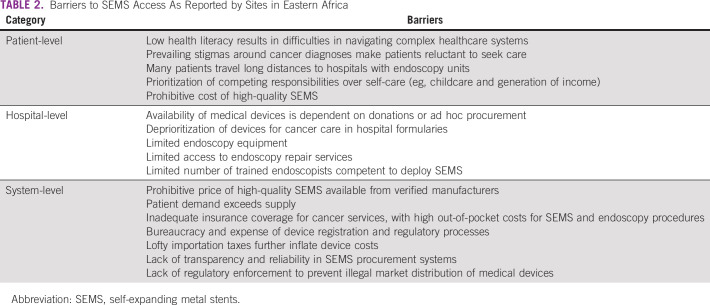
Barriers to SEMS Access As Reported by Sites in Eastern Africa

### Step 2: Analysis of Market Demand

In partnership with CHAI, we conducted a market analysis. First, we attempted to assess the volume of SEMS already available in the region to create an estimate of consumption-based demand; however, this was limited by the fact that SEMS have historically been available only by donations and ad hoc procurement.

We estimated the maximum need for SEMS among patients in the four participating countries to calculate a maximum morbidity-based estimate of demand. Using the reported age-standardized incidence rate of 8.3 per 100,000 population,^16^ we estimated that 12,000 new EC cases occur in these four countries each year. Based on the experience at Tenwek Hospital, we projected that 80% of these patients could potentially benefit from SEMS placement. Therefore, the maximum total market size was projected to be 9,600 SEMS per year, if all patients newly diagnosed with unresectable EC in the four countries were to seek this form of care.

We realize that, however, not all patients who develop EC seek care at a facility with endoscopic capacity. To address this, we also surveyed available literature and databases from National Referral Centers in the four countries for data on their actual case volumes and endoscopic capacity to estimate real-world market demand (Table 1). Based on these results and the actual volume of SEMS used in Kenya and Malawi at the time, we projected that 3,000 SEMS per year would be necessary to meet demand from the participating endoscopy units.

Finally, we calculated a target price of $100 in US dollars at which the out-of-pocket cost for SEMS would be considered affordable for a majority of patients. This was based on expert opinions rendered by health-care workers and hospital administrators in the participating countries.

### Step 3: Selection of an Industry Partner

Concurrent with step 2, we evaluated existing global manufacturers of SEMS with interest and capacity to supply high-quality SEMS at the target price point. In partnership with CHAI, potential suppliers of esophageal stents that met metrics of both quality and affordable pricing within the regional context were identified. To accomplish this, we identified the stringent regulatory agencies (SRAs) that perform quality assessments of medical devices, including SEMS, and determined that approval by one international SRA was sufficient for inclusion as a potential supplier. Either a device approval by the FDA or a CE designation by the European Union was considered satisfactory.

A one-page summary of the initiative was distributed to each manufacturer. Representatives from CHAI and AfrECC engaged in assessments with all respondents to comprehensively evaluate (1) regulatory status of pertinent devices, (2) technical specifications of the devices, (3) pricing and willingness to consider discounted pricing in a developing market, and (4) forecasts for production capacity. The Comprehensive Review of Esophageal Stents was reviewed to identify manufacturers of stents with pre-existing SRA approval for further inquiry.^5^

A total of 16 international manufacturers were identified in China (n = 8), South Korea (n = 3), the United States (n = 3), the Czech Republic (n = 1), and India (n = 1). In engaging with manufacturers, CHAI considered International Organization for Standardization^6^ certifications, the presence in international markets, and existing regulatory approvals. In May 2018, Boston Scientific Corporation (BSC, Marlboro, MA) formally announced its commitment to collaborate with AfrECC and CHAI to launch an access program to provide esophageal SEMS to patients in participating countries at a subsidized price.

### Step 4: Establishment of Regulatory and Procurement Processes

In Kenya, Tanzania, Malawi, and Zambia, we undertook parallel efforts to identify the national regulatory bodies that oversee ethical processes for (1) the donation of medical devices for training purposes and (2) the registration and ongoing procurement of medical devices. We initiated in-person meetings with regulatory representatives in each country. For many device manufacturers without an already established presence in Eastern African markets, the complexities of navigating regulatory bodies, fees, and nuances were prohibitive. Perceptions that the region presents a difficult investment environment because of lax enforcement of procurement conduct are pervasive and may impede the development of reliable supply chains.^17^

Because regulatory and procurement processes for medical devices are administered by national governments, we observed wide variations across the four countries. Regulatory processes in Kenya and Tanzania were nuanced and extensive, requiring registration, submission of a dossier, and payment of registration and inspection fees. Navigating device registration in these countries was expensive and time-consuming, resulting in delays in establishing a procurement process. By contrast, device registration was not required in Malawi and Zambia. At the time of this publication, BSC SEMS were officially sanctioned as registered medical devices in all four countries. Following registration, procurement processes must also be established with individual hospitals.

### Step 5: Development of Endoscopic Training Resources

SEMS deployment requires advanced endoscopy skills for complex procedures and knowledge to manage potential adverse events.^18-20^ Thus, we sought to develop standardized training processes to achieve proficiency in SEMS deployment for participating endoscopists. A curriculum to train endoscopists and ancillary support teams, including anesthetists and nurses, was developed in collaboration with BSC and the American Society for Gastrointestinal Endoscopy (ASGE).

Trainings were led by experienced endoscopists and BSC representatives to transfer procedure-specific and device-specific knowledge, respectively. We employed the Training-of-Trainers (ToT) model to ensure that training resources within Africa would be sustainable and could expand to meet demand without dependence on international trainers. In the initial phase, trainers included a combination of African and US-based endoscopists with extensive experience with SEMS placements. Trainees were selected based on prespecified criteria (Table 3), with expectations that they would become future trainers.

**TABLE 3:**
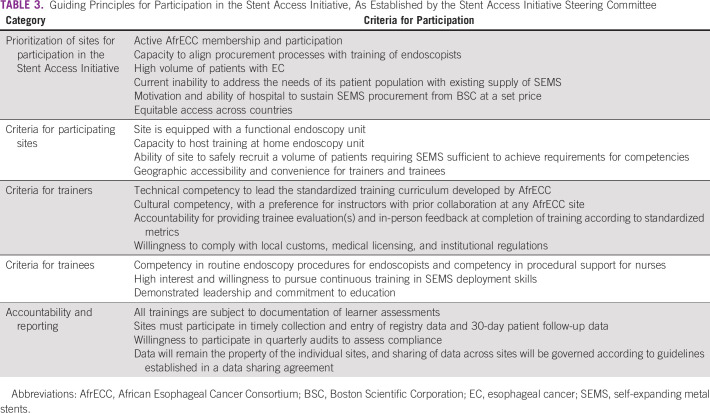
Guiding Principles for Participation in the Stent Access Initiative, As Established by the Stent Access Initiative Steering Committee

The training consisted of a modular didactic curriculum and hands-on training. Trainees were evaluated based on observation, using a standardized competency evaluation form with set metrics for every procedure. A trainee's progress toward becoming a trainer was also measured through serial evaluations. Graduated trainees were deemed to be trainers based on the demonstrated procedural proficiency, successful management of procedural complications, and ability to train others. The duration of each training was 1 week, and the ratio of trainees to trainer was 2:1 to maximize hands-on experiences and ensure vigilant supervision. Details of the training curriculum will be published separately.

### Step 6: Creation of a Device Registry

We designed and implemented a registry to collect standardized data on SEMS utilization at all sites. This registry was informed by the experience of the Surgical Implant Generation Network Fracture Care International, which employs a centralized database to collect data on trauma cases in low- and middle-income countries that require the use of their implants.^21^

We engaged key stakeholders, including clinicians, hospital leaders, and representatives from BSC, to identify key priorities for data collection. Five themes emerged as consensus priorities including (1) patient safety, (2) quality improvement, (3) monitoring endoscopists for procedural competency, (4) supply chain management (eg, forecasting future needs), and (5) diversion avoidance (eg, to ensure that none are diverted for export or commercial sale). Additionally, as the scope of data collection was defined, several prioritized metrics mirrored key procedural steps in stenting (eg, measurement of tumor site from incisors and gastroesophageal junction), and it was recognized that routine data collection could be an effective strategy to reinforce technical principles. Prospective assessment of 30-day patient outcomes was adopted as a key outcome metric.

The SEMS registry database was developed using REDCap, a secure web-based application. The registry captures the following data for every SEMS placement from participating sites: patient demographics, procedural information, serial numbers of SEMS, clinicopathologic data, immediate adverse events, postprocedural quality of life metrics, and 30-day outcomes. Sustainability of the registry will be evaluated on an ongoing basis with assessments of completeness of data entry at each site. Data sharing agreements are currently under discussion with participating institutions. We intend to publish the findings from the AfrECC Stent Registry in subsequent manuscripts, as either single institution case series or multicenter publications.

### Step 7: Establishment of Principles of Accountability and Transparency

As public awareness of the initiative to improve access to SEMS increases, health-care systems will likely face increased demand for SEMS. In preparation for broader dissemination of SEMS into markets in Eastern Africa, a SEMS Access Initiative Steering Committee (SAISC) was formed, composed of one representative from each of the four participating countries. The SAISC was charged with the task of establishing guiding principles to ensure transparency and to promote accountability for participation in the initiative (Table 3).^22^ Transparent distribution of SEMS and training activities among participating countries will be maintained through equitable selection of participating sites, trainers, and trainees, based on established criteria. Moreover, the SAISC will aim to address the sustainability of the initiative through requirements for accountability, stating that continuous provision of subsidized stents is contingent on the timely reporting of data to the SEMS registry.

## DISCUSSION

Within numerous countries in Eastern Africa, EC ranks as a leading cause of cancer-related death. Esophageal obstruction, dehydration, and malnutrition are common presenting features, and palliation is the mainstay of care for a vast majority of patients.^23^ Despite the tremendous burden of EC in Eastern Africa, SEMS remain inaccessible to many African patients because of myriad barriers. To address this need, we forged a partnership with stakeholders from industry, the nonprofit sector, academic institutions, and government agencies to establish sustainable supply chains for a much-needed medical device. Although this partnership has resulted in expanded access to SEMS for EC palliation, we have encountered several challenges that should be highlighted.

Once criteria for quality and affordability were applied, potential industry partners were limited. Many potential suppliers were reticent to enter into emerging markets in Eastern Africa because of concerns regarding corruption, too much or too little regulatory oversight, and an inability to produce medical devices at a cost that was affordable for markets in Eastern Africa. Nonetheless, there was genuine interest expressed by several companies, and we were successful in identifying an industry partner with beneficent interests in improving patient access and introduction into an emerging market.

Ideally, the process of improving SEMS access would be based on a rigorous analysis of regional demand; however, this analysis was limited by the paucity of reliable cancer registries in the region and the likelihood that many cancer cases were not documented. As a result, our analysis of market demand was largely based on real-world estimates. We anticipate that demand may increase as access to cancer therapies improves within the region and as more patients seek care. We acknowledge that the volume of SEMS available from a single manufacturer may become a limitation in the future, if demand exceeds capacity. To date, this has not been an issue, and we are working to project future demand volumes on an annual basis.

According to WHO's principles of good governance and good regulatory practice, the effective and efficient regulation of medical products must account for national health plans, existing laws, available resources, and production and importation practices.^24^ Manufacturers of medical devices are held to high standards because of the potential severity of the consequences of introducing inferior or unsafe products to the marketplace.^25^ Interpretations of these principles were highly variable across national governments; however, our attempts to navigate regulatory and procurement permissions with national regulatory bodies in Tanzania and Kenya revealed nuanced procedures and high fees. In Zambia and Malawi, regulatory processes were less stringent by comparison. The challenges of pursuing regulatory approvals for the same medical device in four countries highlight the need for consistent and transparent oversight of device registration processes, particularly in developing markets. Across all sites, navigation of regulatory bodies to ensure that access to SEMS is nationally sanctioned was critical to addressing system-level barriers to access. However, the formation of partnerships to address the system-level barriers was alone insufficient, and dedicated efforts to address hospital-level barriers are ongoing.

To date, we have primarily focused on the largest referral hospitals in the four countries; however, we are aware that this is not representative of the full geographic scope of demand as many patients may not pursue care beyond smaller regional hospitals. Training of competent endoscopists and ancillary support teams is critical to ensuring safe introduction of a new medical device. Thus, with the help of BSC and the ASGE, we have developed a training program, including a standardized curriculum and assessments of the trainees. We have employed an innovative ToT approach, with the aim of rapidly scaling up training for endoscopists and staff at additional sites.

Finally, coordinated stewardship of this initiative by the SAISC will be critical to upholding guiding principles of transparency and accountability, with the aims to ensure that (1) patients with EC have timely access to and receive quality and affordable SEMS, (2) SEMS are supplied in an equitable and transparent manner across participating sites, (3) SEMS are deployed responsibly with set accountability measures to limit adverse events, and (4) the SEMS registry is maintained as a data repository for monitoring and reporting on safety issues and avails collated data for guideline and policy development. A mandate for sites to participate in the registry will serve to provide monitored channels of SEMS distribution in the region; however, meeting these requirements may be obfuscated by limitations in human resources for data entry, by institution-specific policies regarding data sharing, and by different models of SEMS distribution within each country.

In conclusion, we share this collaboration between AfrECC, CHAI, BSC, the ASGE, academic partners, and local stakeholders as an example of a multisector partnership formed to provide an innovative solution to align regional needs with a supply chain for a high-priority medical device. Progress in closing the divide of availability of essential medical devices for patients with cancer is overdue. Defining global priorities is an important first step undertaken by the WHO;^26^ however, there is a need for medical device prioritization to be further refined to align with local needs. Implementation strategies will require thoughtful coordination and formation of partnerships between industry, academia, health-care systems, and policy makers.
